# Prediction of state anxiety by machine learning applied to photoplethysmography data

**DOI:** 10.7717/peerj.10448

**Published:** 2021-01-15

**Authors:** David Perpetuini, Antonio Maria Chiarelli, Daniela Cardone, Chiara Filippini, Sergio Rinella, Simona Massimino, Francesco Bianco, Valentina Bucciarelli, Vincenzo Vinciguerra, Piero Fallica, Vincenzo Perciavalle, Sabina Gallina, Sabrina Conoci, Arcangelo Merla

**Affiliations:** 1 Department of Neuroscience, Imaging and Clinical Sciences, Institute for Advanced Biomedical Technologies, University of Chieti-Pescara, Chieti, Italy; 2 Physiology Section, Department of Biomedical and Biotechnological Sciences, University of Catania, Catania, Italy; 3 Institute of Cardiology, University of Chieti-Pescara, Chieti, Italy; 4 STMicroelectronics, ADG R&D, Catania, Italy; 5 Department of Sciences of Life, Kore University of Enna, Enna, Italy; 6 Department of Chemical, Biological, Pharmaceutical and Environmental Science, University of Messina, Messina, Italy

**Keywords:** Photoplethysmography (PPG), Machine learning, General linear model (GLM), State-trait anxiety inventory (STAI-Y), Affective computing

## Abstract

**Background:**

As the human behavior is influenced by both cognition and emotion, affective computing plays a central role in human-machine interaction. Algorithms for emotions recognition are usually based on behavioral analysis or on physiological measurements (e.g., heart rate, blood pressure). Among these physiological signals, pulse wave propagation in the circulatory tree can be assessed through photoplethysmography (PPG), a non-invasive optical technique. Since pulse wave characteristics are influenced by the cardiovascular status, which is affected by the autonomic nervous activity and hence by the psychophysiological state, PPG might encode information about emotional conditions. The capability of a multivariate data-driven approach to estimate state anxiety (SA) of healthy participants from PPG features acquired on the brachial and radial artery was investigated.

**Methods:**

The machine learning method was based on General Linear Model and supervised learning. PPG was measured employing a custom-made system and SA of the participants was assessed through the State-Trait Anxiety Inventory (STAI-Y) test.

**Results:**

A leave-one-out cross-validation framework showed a good correlation between STAI-Y score and the SA predicted by the machine learning algorithm (*r* = 0.81; *p* = 1.87∙10^−9^). The preliminary results suggested that PPG can be a promising tool for emotions recognition, convenient for human-machine interaction applications.

## Introduction

Emotions play a fundamental role in human life, since they affect both human physiological and psychological status. Positive emotions are often related to an improvement of human health and work efficiency, whereas negative emotions may provoke health problems such as depression (Cummings, Caporino & Kendall, 2014).

Given the recent proliferation of human-machine interaction applications (e.g., marketing, automotive, teaching, entertainment (Broekens, Heerink & Rosendal, 2009; Kirby, Forlizzi & Simmons, 2010; Bota et al., 2019; Filippini et al., 2020)), the study of human emotions gained relevant attention. For example, automotive systems, are useful to monitor the mental state of the driver and may be used to keep him alert during driving, reducing car accidents (Schuller & Rossion, 2004). Moreover, studying the emotion raised by a call center conversation may help to improve quality of service of a call attendant (Chul Min & Narayanan, 2005). Furthermore, medical doctors could exploit the emotional contents of a patient’s speech to diagnose various disorders (France et al., 2000). In addition, human behavior and cognitive performances are deeply influenced by emotions (Ochsner, Silvers & Buhle, 2012; Costantini et al., 2013). Among the emotions that could affect the human behavior, anxiety can produce negative effects (Eysenck & Calvo, 1992; Eysenck et al., 2007; Perpetuini et al., 2018, 2019a). Anxiety can be defined as a biological warning system that alerts the body to react mentally and physically to potentially aversive stimulations. In normal condition, anxiety produces the increase of muscle tension and activates mostly the sympathetic nervous systems. This emotion provokes negative effect on visual attention (Janelle, 2002) and cognitive performances (Eysenck et al., 2007), thus it could be fundamental to measure the level of anxiety during activities which exploit cognitive functions. Usually, the level of anxiety is measured by administering psychological tests; however, in some applications, an interaction between clinician and subject is not allowed (e.g., automotive systems), thus a model based on physiological signals is needed.

Generally, this kind of models rely on physiological measurements, assessing heart rate, skin conductance, skin temperature, blood pressure, pupil size, and brain activity (Tan & Picard, 2007; Jerritta et al., 2011). Among these measures, the heart rate variability (HRV) was proven to provide salient information regarding the psychophysiological condition of a subject (Thayer et al., 2012; Shaffer & Ginsberg, 2017; Kim et al., 2018). One approach to estimate HRV is to analyze the temporal variation of the highest peaks (R-peaks) repetition rate in the electrocardiography (ECG) trace, which are indicative of the ventricular depolarization. As an example, the root mean square of the successive differences (RMSSD) of the neighboring ECG R-peaks (R–R intervals) is a time domain metric considered to be sensitive to the autonomic nervous system’s parasympathetic branch (Sollers et al., 2007). In the frequency domain, the ratio between power spectrum densities (PSDs) at low-frequency (LF, 0.03–0.15 Hz) and high frequency (HF, 0.15–0.35 Hz) bands of the HRV is commonly employed (Malliani, Lombardi & Pagani, 1994). Indeed, HF is indicative of the parasympathetic system activation, whereas LF is sensitive to both the sympathetic and parasympathetic branches, hence, their ratio (LF/HF) is suggestive of the balance between the two systems (Malliani, Lombardi & Pagani, 1994; Malik et al., 1996; Barold, 2003).

Photoplethysmography (PPG) is an optical technique able to estimate HRV by means of the Pulse Rate Variability (PRV) (Longmore et al., 2019). PPG is sensitive to hemoglobin oscillations in tissues, which are associated to volumetric modulations of peripheral arteries induced by the propagation of pulse pressure wave from the heart (Allen, 2007). PPG probes are usually equipped with a source, injecting near infrared (NIR) light into the tissue, and a photodetector, gathering the re-emitted light. PPG measurement can be performed in two main modalities: in transmission modality the source and the detector are placed on two opposite surfaces of the same body district, whereas in back-reflection modality the source and the detector are placed on the same surface at a few centimeters distance (Allen, 2007). Thanks to the high diffusive properties of biological tissue in the near infrared spectral range, the back-reflection modality allows to record signal from large body sites and deep (few centimeters from the skin) arteries, such as tibial and brachial arteries. Since PPG is non-invasive, cheap and easy to use (do not require specialized operators), this technique is widely employed in clinical practice. For instance, PPG is commonly used to monitor oxygen saturation on fingers or earlobes using multi-wavelengths recordings. Moreover, single-wavelength PPG can also evaluate arterial stiffness (Chiarelli et al., 2019a; Perpetuini et al., 2020) assessing PPG shape and speed of propagation of the pulse wave (e.g., Pulse Wave Velocity, PWV) at multiple body locations (Nandini & Pandey, 2018). Furthermore, PPG signal is associated to intravascular pressure and several models have been proposed to predict blood pressure from PPG (Chandrasekaran et al., 2012; Ding et al., 2015; Lazazzera, Belhaj & Carrault, 2019). Since PPG signal can provide information of PRV (Jeyhani et al., 2015) and blood pressure, which are modulated by autonomic activity, PPG features might be indicative of the psychophysiological status of the subject. Several studies investigated the relationship between PPG and stress, often combining this technique with other technologies acquiring heterogeneous physiological signals (Cho, Julier & Bianchi-Berthouze, 2019; Akbar et al., 2019).

The aim of this study was to develop a model to estimate the state anxiety (SA) of subjects relying on PPG features.

Although different models that link PPG features with quantitative physiological parameters (e.g., blood pressure (Lazazzera, Belhaj & Carrault, 2019)) are available, the elaboration of an explicit and direct model linking PPG to SA has remained a difficult task. In the present study the prediction of SA was generated employing a data-driven multivariate statistical procedure (i.e., machine learning approach) applied to multiple PPG-driven estimates of blood pressure and PRV metrics. This method did not require inference of the physiology of the system, but the only a-priori hypothesis was the presence of a physiological link between emotional condition and PPG. This assumption was tested a-posteriori with the assessment of generalization performance of the model. In particular, the multivariate relationship between PPG features and SA was investigated employing the General Linear Model (GLM) in a supervised and cross-validated learning framework (Monti, 2011; Chiarelli, Romani & Merla, 2014; Perpetuini et al., 2019b). Notably, the multivariate approach used provided a single dependent variable and multiple independent features. Finally, a Receiver Operating Characteristic (ROC) (Zweig & Campbell, 1993) analysis was performed to test the capability of the predicted SA form PPG features to discriminate high contingent SA as described by the State-Trait Anxiety Inventory (STAI-Y) (threshold = 40) (Rinella et al., 2019). It is worth to notice, that the novelty of this work does not rely on the specific statistical algorithm used (i.e., multivariate regression), but it is based on the employment of a multivariate algorithm for prediction of SA from several PPG features in a machine learning framework, which is a general field of applied statistics that uses multivariate approaches for prediction purposes (Michie, Spiegelhalter & Taylor, 1994). The goal of this classification procedure is to have information concerning SA of a subject. This model is useful in applications where the anxiety could affect human cognitive performances (e.g., automotive applications, brain-computer interface, clinical practice) but a direct interaction with a psychologist is not allowed, thus making impossible the administration of psychological tests, such as STAI-Y.

## Materials and Methods

### Participants

A total of 102 volunteers (Male/Female: 54/48, age ranging from 20 to 70 years old, mean age: 34.3 years, standard deviation: 15.5 years) participated in the study. All subjects were requested to not take medications, coffee or energy drinks before the experiment. All the participants signed informed consent and could withdraw from the study at any time. The measurements were performed in agreement with the ethical standards of the Helsinki Declaration and approved by The Comitato EticoCatania 1 (authorization n. 113/2018/PO). Demographic information, such as age and gender, were gathered from all the participants.

### Photoplethysmographic measurements

**Figure 1:**
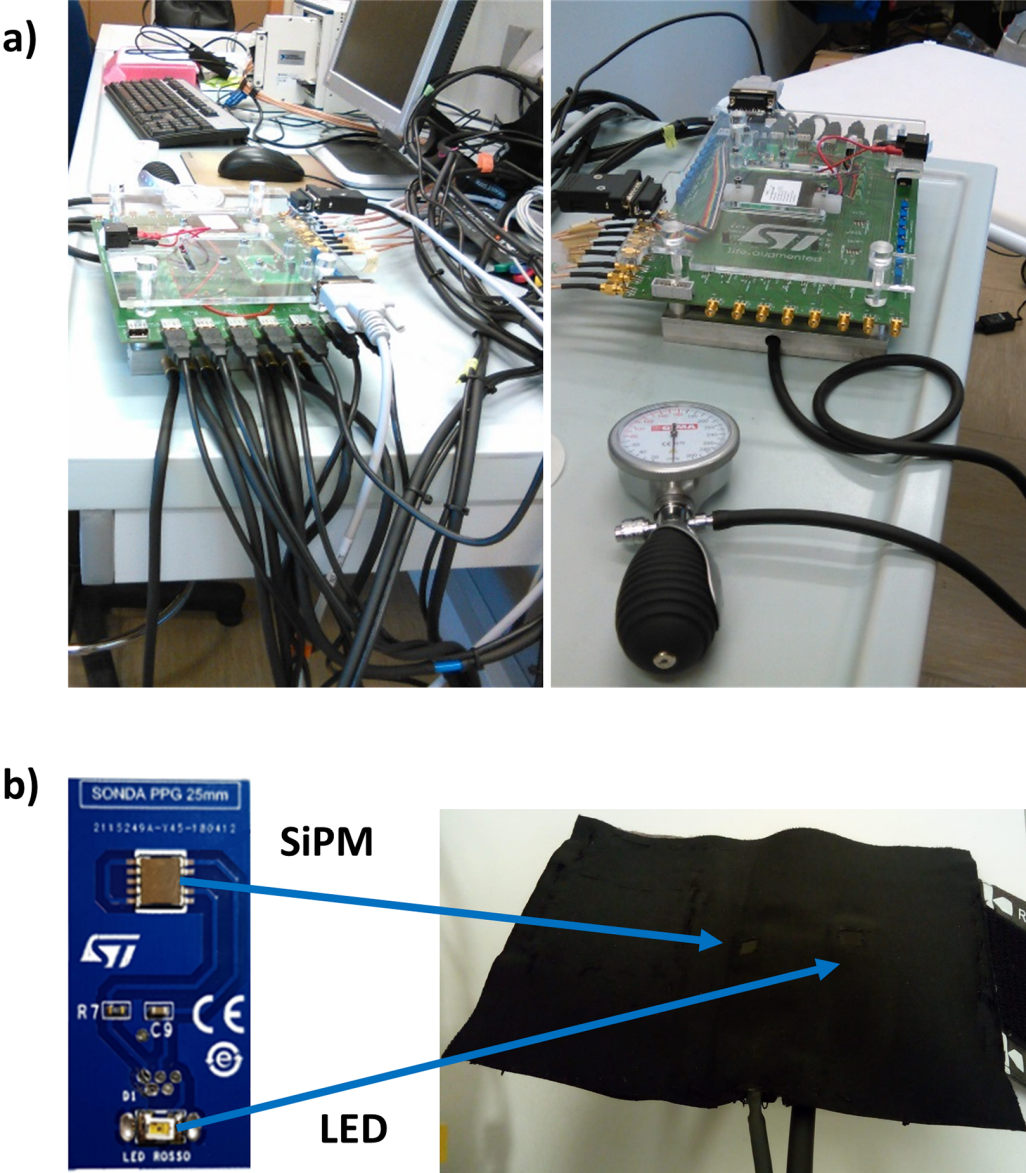
(A) PPG system employed; (B) PPG probe were made of a LED at 940 nm of wavelength and a silicon photomultiplier (SiPM).

Photoplethysmographic was collected employing a home-made multi-channel system (Vinciguerra et al., 2018; Perpetuini et al., 2019c, 2019d) (Fig. 1A) equipped with seven optical probes. As previously described in Perpetuini et al. (2020), the optical probes were developed by STMicroelectronics (Catania, Italy) and they were composed by a light source consisting in a Light Emitting Diode (LED) emitting a wavelength range centered at 940 nm (SMC940 LED, Roithner Laser Technik, Vienna, Austria) coupled with a detector composed of Silicon PhotoMultiplier (SiPM) chip (Vinciguerra et al., 2017). Light source (LED) and detector (SiPM) were located at an inter-optical distance of 4 cm. Each probe (Fig. 1B) was placed on a bracelet equipped with a pressurized cuff insufflated to an under-diastolic pressure of 60 mmHg in order to not affect the pulse waveform. The sampling frequency of the PPG channels provided by home-made multi-channel system was set at 1 kHz. Thanks to the high sensitivity of the SiPM detector, the probe was able to acquire signals in the near infrared spectral range coming from deep arteries in a back-reflection modality.

A total of 2 out of the 7 available probes were employed in the study and they were placed on the right brachial and radial arteries. The choice of using only 2 probes was driven by the idea of performing a non-invasive and fast measurement.

Two different experimental procedures were performed. Concerning the first experiment, 67 subjects were requested to lay supine on a medical cot. PPG data were acquired for 30 s. During the second experimental protocol, an anxiety manipulation was administered to 35 participants. Particularly, participants were asked to stay supine on a medical cot while watching a video selected according to Koelstra et al. (2012). Since the stimulation lasted more than 30 s, in order to compare the data acquired between the two experimental protocols, only the last 30 s of the data collected during the second procedure were considered in the data analysis.

### Photoplethysmographic signal pre-processing

The signal pre-processing was performed in accordance with Perpetuini et al. (2020). Specifically, raw PPG signals were converted into optical densities (ODs) (Chiarelli et al., 2019b). The ODs were downsampled of a factor 10 (down to 100 Hz), then filtered with a zero-lag, 4th order, band-pass Butterworth digital filter (cut-off frequencies: 0.2 and 10 Hz). The PPG peaks were identified considering the local maxima on the filtered and normalized (*z*-scored) signals. In order to improve the accuracy of the procedure, some constraints were defined: based on visual inspection, the minimum value of the peak of the normalized signal was set at 2 and the temporal interpeak distance was set at 600 ms, which is compatible with the human heart rate at rest. A visual inspection showed a 100% accuracy of the method. The peaks identification allowed to evaluate the different metrics associated to PRV and its PSD (Parhi & Ayinala, 2014) that were used as predictors in the model.

**Figure 2:**
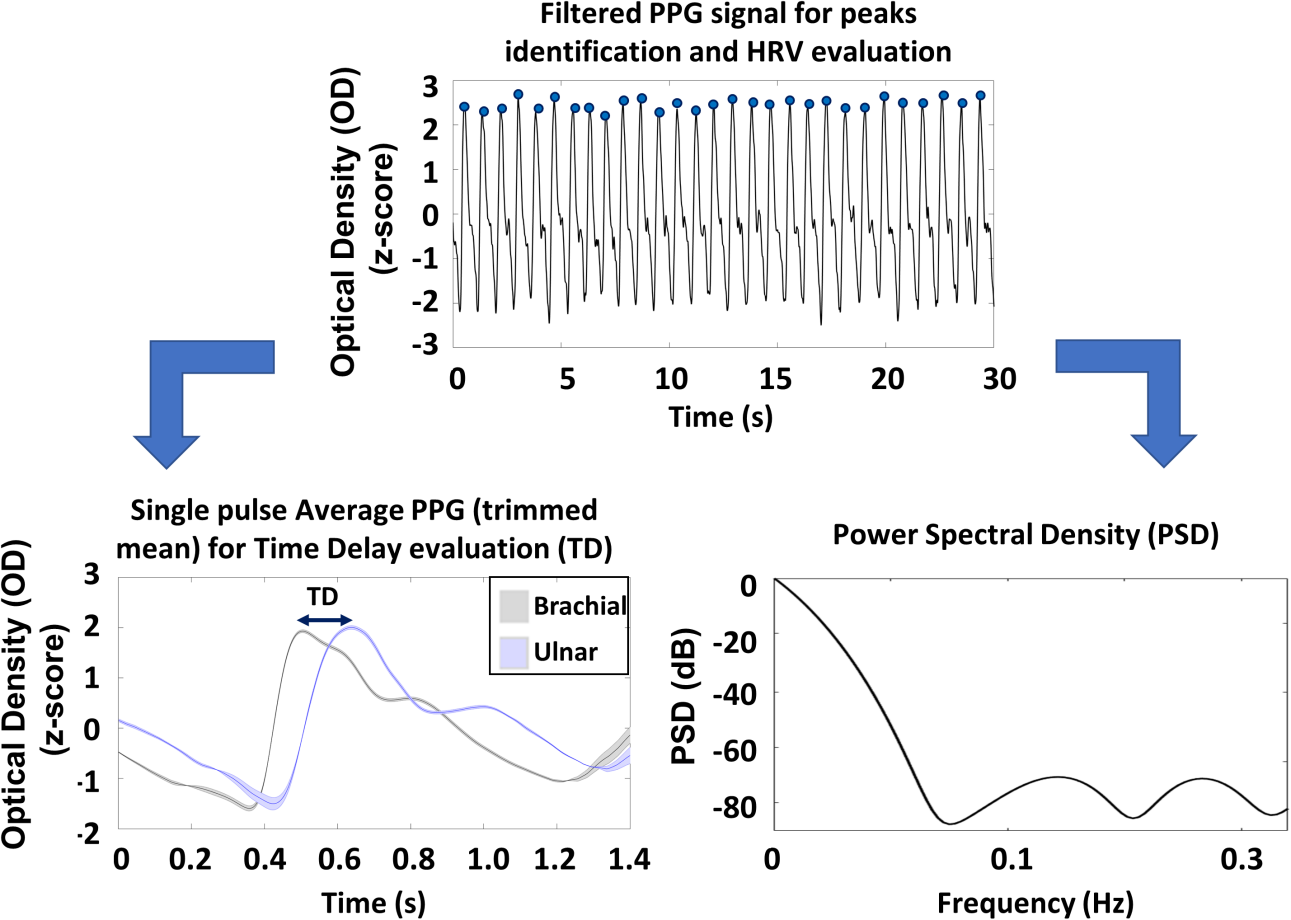
Schematic representation of the preprocessing steps of PPG signals and an example of the pulse average PPG with associated standard errors (shaded areas).

Single pulse PPG was evaluated by averaging the signal in a time window from 0.6 s prior to 1.2 s after the systolic peak of the PPG ulnar signal. The averaging procedure was performed employing a trimmed mean approach, considering only single pulses with all the values comprises between the 25th and 75th percentile of the sample population, making the estimation of the pulse average PPG more stable. The single pulse evaluation allowed to assess the average time delay (TD) between the PPG peaks collected at the ulnar and brachial arteries. In Fig. 2 the preprocessing steps are reported together with an average single pulse PPG and associated standard errors (shaded areas).

### State anxiety evaluation and prediction through general linear model

The SA of the participants was assessed administering the STAI-Y (Spielberger, 2010), which is one of the most common and sensitive measure of anxiety in applied psychology research (Marteau & Bekker, 1992). STAI-Y is a psychological inventory based on a 4-point Likert scale, 1 (not at all) to 4 (extremely), and consists of 40 questions on a self-report basis (Spielberger et al., 1983). STAI measures two types of anxiety: SA, or anxiety about an event; and trait anxiety, or anxiety level as a personal feature. Higher scores are positively correlated with higher levels of anxiety. Its most common revision is Form Y. For this study only SA was evaluated, in order to obtain information about participants’ anxiety related to experimental setting. The test was administered immediately after the experimental session. It is fundamental to highlight that STAI-Y does not necessarily measure anxiety produced by the experimental setting, but it is indicative of anxiety in that specific moment that could be dictated by the experimental settings and/or external factors. However, the aim of this work was to estimate SA from PPG features, hence knowing the cause of the anxiety is not mandatory, since an altered SA influences both STAI-Y questionnaire results and the physiology of the subject.

A machine learning approach was applied training a GLM on the SA evaluated by the STAI-Y through a supervised learning approach.

GLM is a statistical linear model that can be expressed as described in Monti (2011):

(1) $$Y = X {\rm{\beta}} + {\rm{\varepsilon}}$$

where:

- *Y* = *n* × 1 column vector describing the dependent variable (e.g., SA)
- *X* = *n* × *p* design matrix: *p* regressors are reported in columns of length *n* (e.g., PPG features);
- β = *p* × 1 column vector of weights of each predictor;
- ε = *n* × 1 column vector of the residual error

During the training procedure, the parameters βs are optimized, generally employing a least square approach. The regressors and the dependent variable are usually normalized. In this case, βs are representative of the strength of the association with *Y*.

The following 4 independent variables (3 of them were extracted from the PPG signals) were normalized (*z*-score) and used for the GLM based estimation:

- Estimated systolic blood pressure from PPG signal acquired on the right arm according to Lazazzera, Belhaj & Carrault (2019) (ABP), evaluated as:

$$\rm {ABP = 184.3 - 1.329\cdot HeartRate + 0.0848*T{D_{ulnar - brachial}}}$$

where $\rm {HeartRate}$ is expressed in beat per minute (bpm) whereas $\rm {T{D_{ulnar - brachial}}}$ is the time delay between the systolic peaks of PPG evaluated at the ulnar and at the brachial arteries and expressed in milliseconds (ms).

- LF/HF ratio evaluated on the PSD of PRV estimated from the brachial PPG signal. The PRV signal was not filtered. The amplitude of the temporal window allowed to preserve both the LF and HF component. The PSD of the signal was evaluated by means of Welch’s method (Parhi & Ayinala, 2014);
- RMSSD: evaluated on the PRV extrapolated from the brachial PPG signal evaluated as follow:

$${\rm{RMSSD}} = \sqrt {{1 \over {\left( {N - 1} \right)}}(\mathop \sum \limits_{i = 1}^{N - 1} ({{\left( {\Delta SP{)_{i + 1}} - {{\left( {\Delta SP} \right)}_i}} \right)}^2}} )$$

where $\Delta {\rm SP}$ is the time distance between two consecutive PPG systolic peaks and *N* is the number of $\Delta {\rm SPs}$ recorded.

- Gender (female participants labeled as “1” and male participants labeled as “2”).

ABP, LF/HF and RMSSD were a-priori selected relying on previous works performed on the topic (James et al., 1986; Piccirillo et al., 1997; Francis et al., 2009).

Particularly, it is worth to notice that, in order to obtain reliable measurements of PRV, recordings of at least 2 min are recommended (Shaffer & Ginsberg, 2017). However, the purpose of this work is to obtain an estimate of the SA in a very short temporal window, in order to employ this method in applications where a real-time assessment of the SA is needed (e.g., BCI, Human-robot interaction, Assistive purposes). For this reason, it was attempted to estimate SA from PPG features employing a limited temporal window (30 s) that allowed to evaluate LF and HF. In order to test the effect of the short recording on the evaluation of the LF/HF parameter, PPG signals from 35 participants were recorded at rest for 2 min and LF/HF evaluated on the first 30 s of the measurements was compared with the same parameter computed considering all the time course with a paired *t*-test. No statistical differences were found (30 s window vs. 2 min window: *t* = 0.752; df = 34; *p* = 0.457). Hence, it is licit to suppose that the limited temporal window used for the recordings poorly affected the classification purposes of this work.

The study sample was divided into two separate datasets: a training set and a test set. The training set was composed of 75 samples whereas the training set of 27 samples. In order avoid a possible effect of the choice of the training and test set on the performances of the regression, 1,000 random combinations of training and test sets were investigated. The mean value of the procedure was used to predict SA (SAp) that was used for further statistical analysis. The comparison between SA and the cross-validated SAp was performed employing the paired *t*-test (Hsu & Lachenbruch, 2007), the correlation analysis (Cohen, West & Aiken, 2014) and the Bland–Altman plot (Bland & Altman, 1986; Dewitte et al., 2002; Krouwer, 2008) between the two variables. This statistical analysis allowed to test the performances of the machine learning approach in the estimation of SA. A comparison between the multiple regression model with nested models was evaluated computing the incremental *R*^2^.

### Statistical analysis

Statistical evaluations for the demographic variables were performed through paired *t*-test and correlation (Spearman) analysis.

A ROC analysis was carried out to investigate the sensitivity and specificity of cross-validated SAp to high contingent anxiety. SA was considered as the gold standard, thus participants with SA > 40 were labeled as high contingent SA subjects (label = 1), whereas participants with SA ≤ 40 were considered with low contingent SA (label = 0), in accordance with Addolorato et al. (1999) and Julian (2011). In the study sample, 45 participants exhibited SA > 40 and 57 participants showed SA ≤ 40. Since the two classes were not perfectly balanced, a bootstrap procedure (10,000 iterations) was performed in order to evaluate the AUC of the ROC curve with balanced classes (Dupret & Koda, 2001). The ROC analysis allowed to assess how the sensitivity and specificity of SAp for high contingent anxiety identification changes by varying the threshold of SAp. The multivariate regression and classification performances of SAp were compared to the univariate outcomes of single PPG features considering the Area Under the Curve (AUC) of the ROC and the correlation coefficients.

## Results

**Figure 3:**
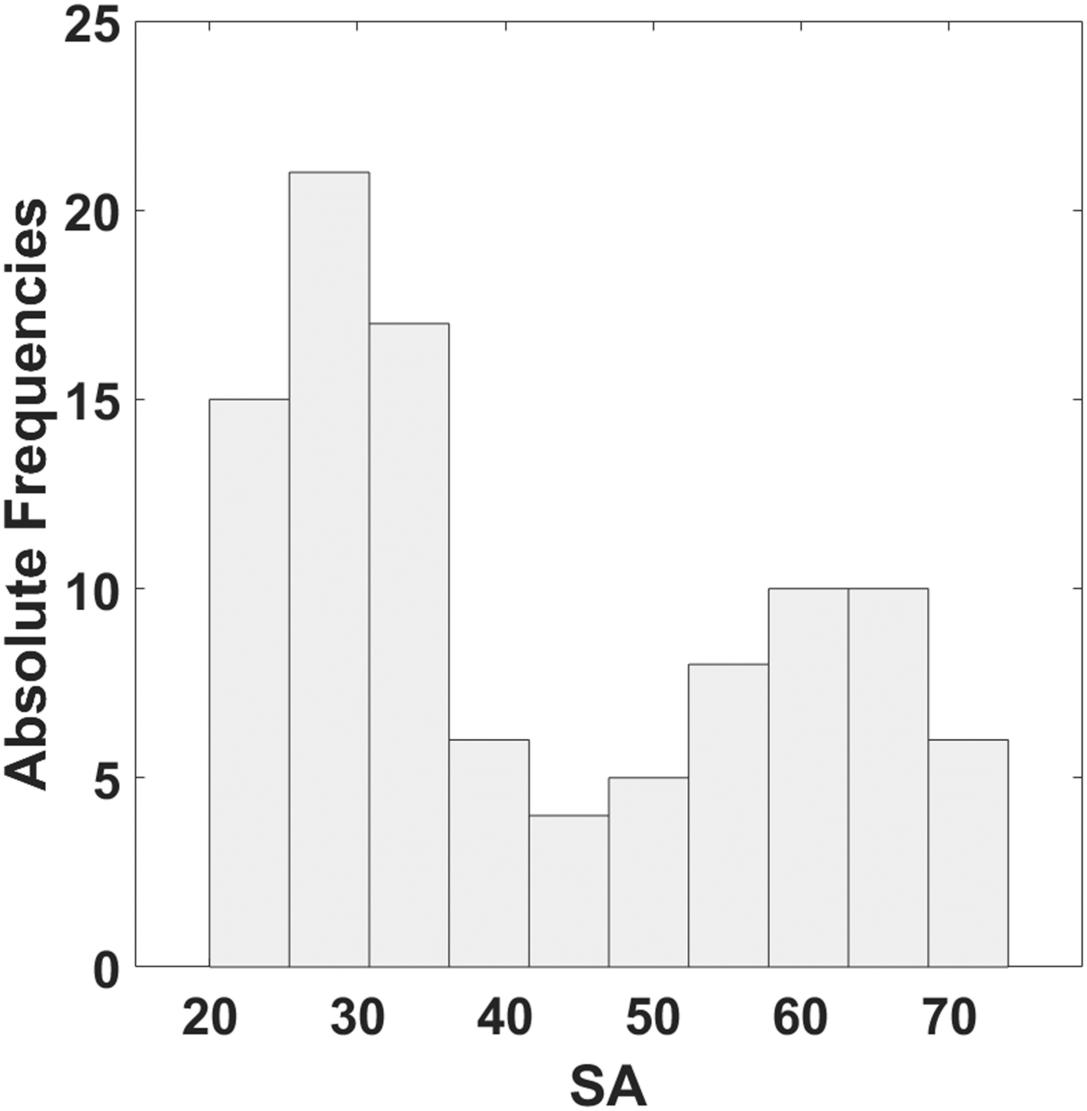
Histogram of the distribution of SA estimated by means of STAI-Y.

The distribution of SA estimated administering the STAI-Y is reported in Fig. 3. The range of SA obtained is between 20 and 74.

No correlation between SA and age was found (*r* = −0.16; *p* = 0.11). Concerning the PPG features, large (Hemphill, 2003) and significant correlation was found for LF/HF (*r* = 0.66; *p* = 4.19∙10^−14^) and RMSSD (*r* = 0.52; *p* = 1.22∙10^−8^), whereas a small significant correlation was found for ABP (*r* = 0.20; *p* = 0.04).

No statistically significant differences concerning the gender of the participants were found for the GLM predictors (males vs. females: ABP: *t* = 0.64, df = 100, *p* = 0.52; LF/HF: *t* = −1.38, df = 100, *p* = 0.17; RMSSD: *t* = −2.64, df = 100, *p* = 0.01). However, significant difference between males and females was found for the SA (males vs. females: *t* = −3.61; df = 100; *p* = 4.77∙10^−4^).

Table 1 reports the average cross-validated GLM βs estimated for each PPG predictor with the associated statistics.

**Table 1:** Average cross-validated β-values and relative *t*-scores and *p*-values estimated through GLM in SA prediction.

Normalized (*z*-scored) regressor	β-value	*t*-stat	*p*-value
ABP	0.2358	3.345	0.001
LF/HF	0.3176	4.080	1∙10^−4^
RMSSD	0.3042	3.544	7∙10^−4^
GENDER	−0.3947	−4.284	6∙10^−5^

**Figure 4:**
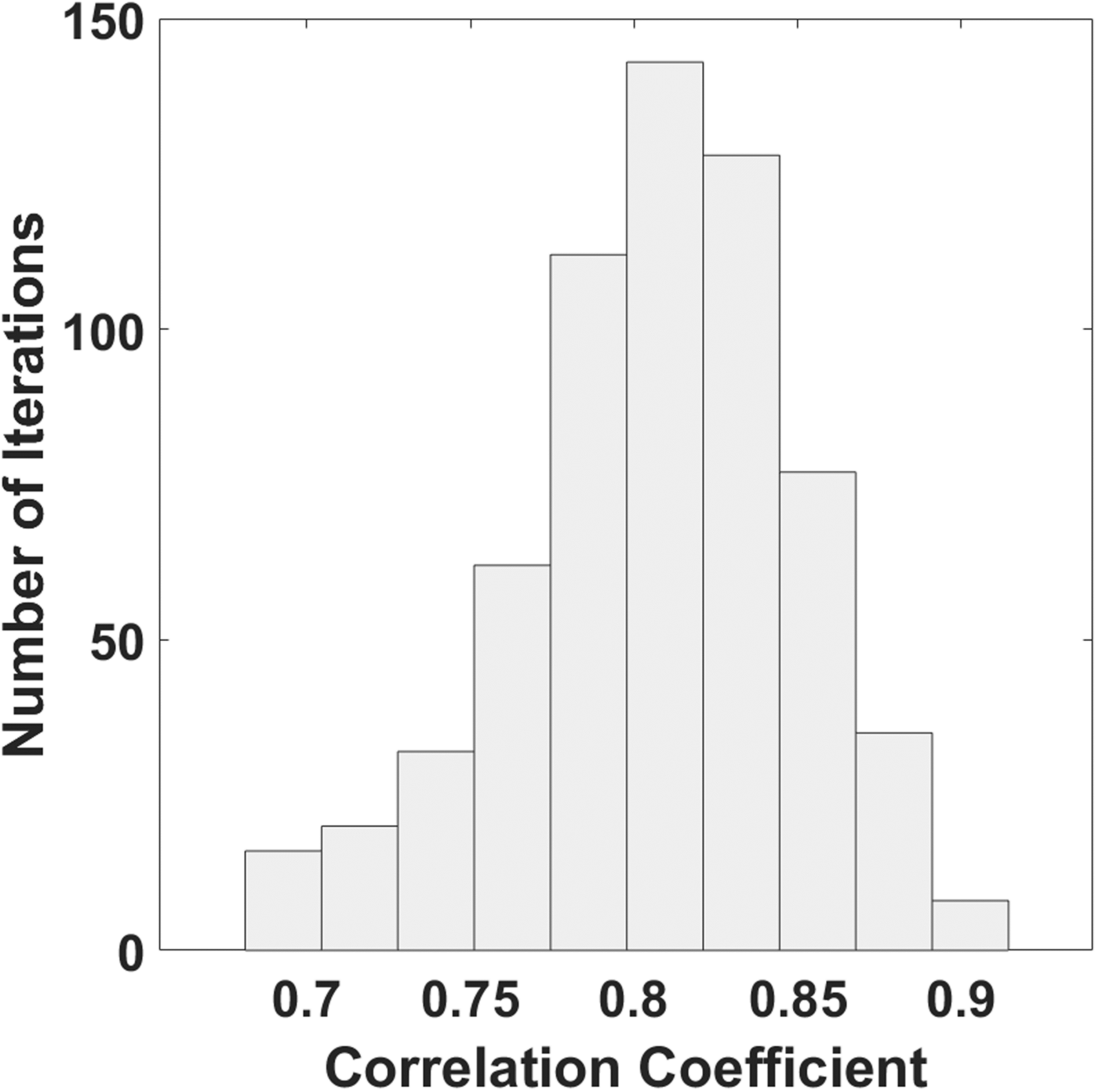
Histogram of the distribution of correlation coefficients estimated with a bootstrap procedure.

Figure 4 reports the distribution of the correlation coefficients obtained with 1,000 combinations of training and test set. The mean value of the correlation coefficient was 0.81, and the standard deviation was 0.05.

**Figure 5:**
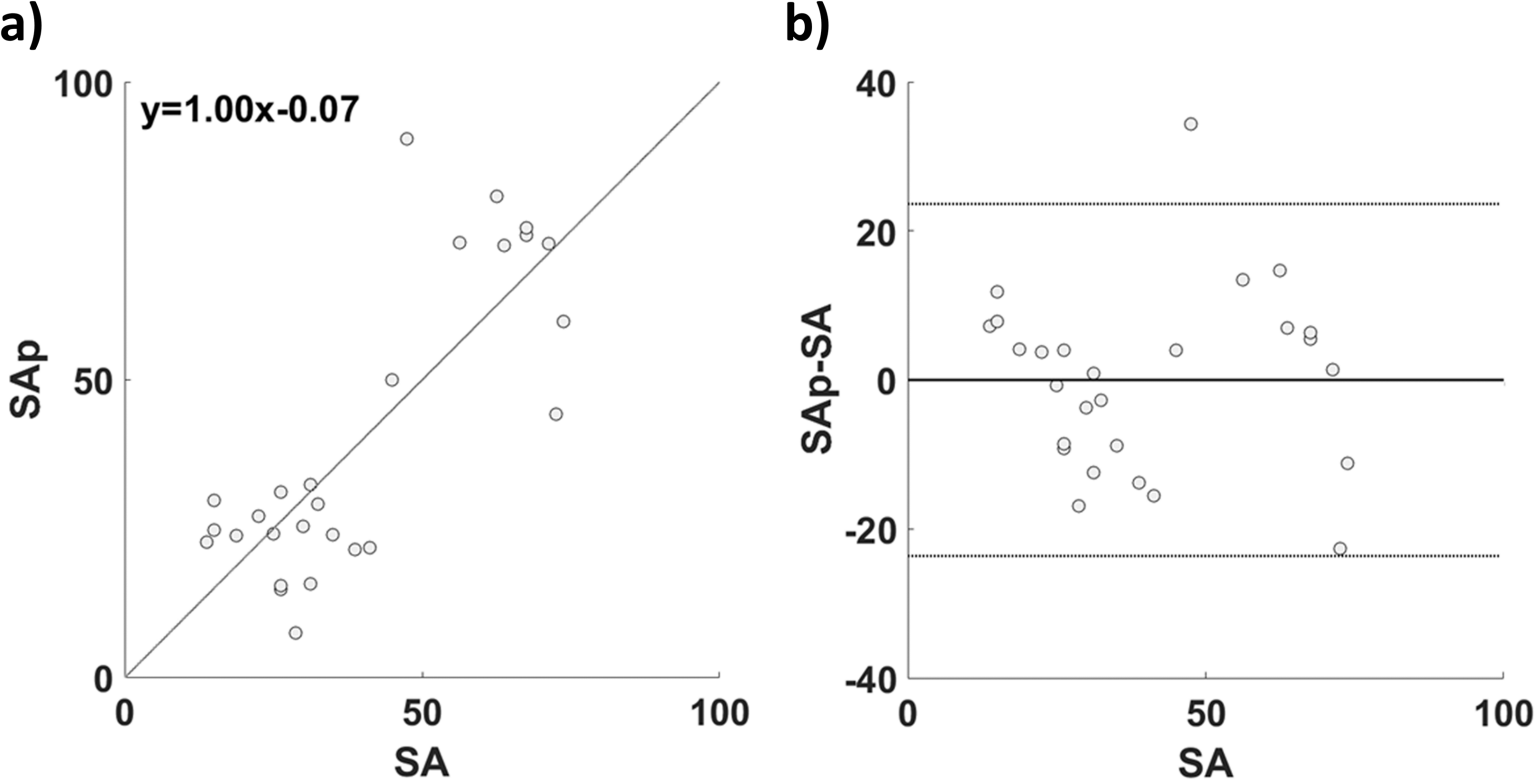
(A) Correlation and (B) Bland–Altman plot of SA and SAp for the GLM based estimation.

Figure 5 reports the comparison between SA and the cross-validated SAp. In Fig. 5A the correlation between SA and SAp (*r* = 0.81; *p* = 1.87∙10^−9^) is reported; in Fig. 5B the associated Bland–Altman plot is shown. The paired *t*-test did not show significant difference between SA and SAp (*t* = 1.56; df = 101; *p* = 0.12).

The univariate correlations between the PPG features used in the GLM and SA were computed and compared to the correlation coefficient (*r* = 0.81) of the multivariate analysis (vs. ABP: *r* = 0.20, *z* = −6.5, p ~ 0; vs. LF/HF: *r* = 0.66, *z* = −2.35, *p* = 0.02; vs. RMSSD: *r* = 0.52, *z* = −3.87; *p* = 1∙10^−4^).

The results of the comparison of the multivariate model with the nested models by means of the evaluation of incremental *R*^2^ with respect to the final *R*^2^ (*R*^2^ = 0.66) is reported in Table 2.

**Table 2:** Incremental *R*^2^ of the nested models with respect to the final multivariate model.

Regressor	Incremental *R*^2^
ABP	−0.07
LF/HF	−0.30
RMSSD	−0.15
GENDER	−0.05

**Figure 6:**
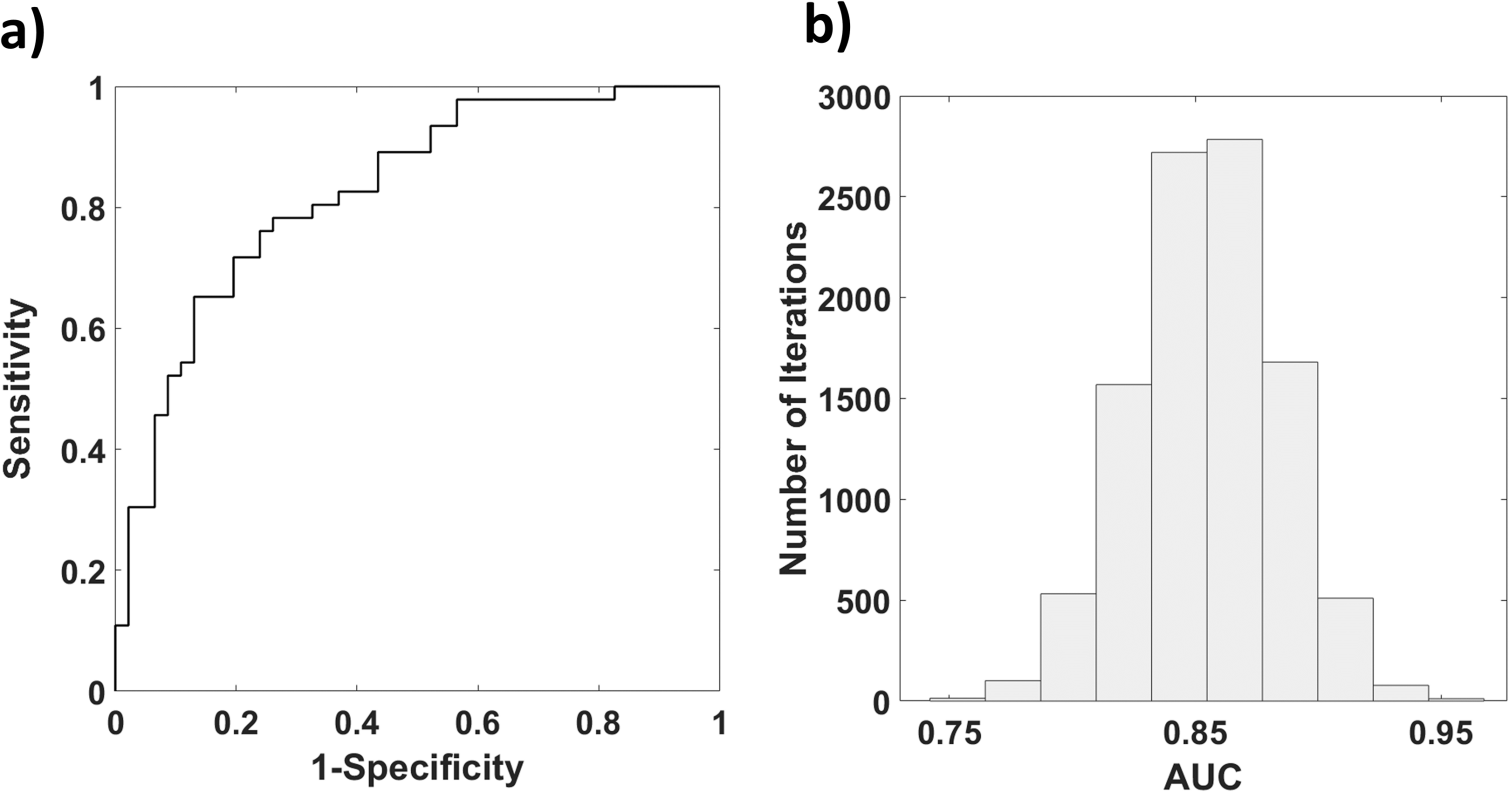
(A) Histogram reporting the distribution of AUC obtained with a Bootstrap procedure; (B) ROC of SAp in assessing high contingent anxiety (SA > 40).

Figure 6A reports the results of the bootstrap procedure for the computation of the ROC of SAp evaluating high contingent anxiety (SA > 40). A ROC AUC of 0.88 was obtained (Fig. 6B).

The AUC found employing the multivariate approach was significantly different (Hanley & McNeil, 1982) from the AUCs obtained from univariate analysis employing the PPG features (ABP: AUC = 0.62, vs. 0.88: *z* = −3.88, *p* = 5∙10^−5^; LF/HF: AUC = 0.78 vs. 0.88: *z* = −1.67, *p* = 0.04; RMSSD: AUC = 0.75 vs. 0.88: *z* = −2.11; *p* = 0.02).

## Discussion

Affective computing and emotions recognition are relevant topics in several applications such as automated driver assistance, healthcare, marketing and teaching (Bota et al., 2019). In human-machine interaction is fundamental to evaluate the emotional compound, since the human behavior depends on both cognitive and emotional features. In this perspective, several models of the human psychophysiological status have been developed, generally based on physiological measurements (e.g., heart rate variability, skin temperature and skin conductance). Among the signals that are indicative of the psychophysiological condition, PPG has the advantage of non-invasiveness, low cost and ease of use, not requiring specialized operators. Since PPG provides an estimation of local blood volume changes in response to the pressure wave propagating in the arterial circulatory system, it encodes information of blood pressure and heart rate, which are metrics highly associated to the psychophysiological status. However, simple models linking PPG to emotional condition are still not available. In this article, the capability to estimate the SA from multiple PPG features in a data-driven framework is reported. A multivariate machine learning approach based on GLM was employed. In the model, the physiological features derived from PPG were used as predictors and the SA, evaluated through STAI-Y, was considered as the dependent variable. A two-fold cross validation (i.e., training set and test set) was employed to test the generalization of the regression. The results confirmed the hypothesis that PPG can be a valuable tool for the prediction of the SA. In fact, a good correlation between SAp, predicted from physiological features derived from PPG and the machine learning model, and SA estimated through STAI-Y was found (*r* = 0.81; *p* = 1.87∙10^−9^). It is worth to notice that the machine learning approach performed better than the univariate regression employing the physiological features derived from PPG, depicting the added value of the multivariate procedure.

The good performance of the model was also confirmed by the Bland–Altman plot, where the errors of the estimation were mostly distributed within the 95% confidence interval (±1.96 standard deviation), showing the solid correspondence of the two methods.

Since differences in SA were found according to gender, a regressor depicting the gender was added in the GLM procedure. The β-value associated to this regressor is different from zero, thus gender participates in anxiety estimation. However, the incremental *R*^2^ relative to gender (*R*^2^ = −0.07), is lower with respect to RMSSD and LF/HF. Concerning ABP, *R*^2^ is similar to that associated to gender, suggesting that the estimation of SA is mainly based on RMSSD and LF/HF. However, the β-value associated to gender is higher with respect to the other regressors, suggesting that it contributes significantly in the estimation of SA.

Finally, a ROC analysis was performed to assess the capability of SAp to classify high contingent SA as depicted by STAI-Y. The multivariate approach exhibited higher performances compared to the univariate classification outcomes obtained using the single GLM regressors, confirming the importance of this method for affective computing applications.

Nonetheless, further studies should be performed to increase the sample size. The Machine learning approach used in this study rely on supervised learning which is inherently a data-driven analysis; data-driven analysis is highly affected by the sample size and the performance of the model could indeed improve reducing a possible overfitting effect driven by the limited sample numerosity. Furthermore, by increasing the sample size, more complex non-linear machine learning models, such as Deep Learning (LeCun, Bengio & Hinton, 2015), could be employed to improve the regression performance of SA from PPG features, also highlighting possible non-linear association among variables.

Moreover, it is worth to highlight that the STAI questionnaire was administered after the PPG data acquisition. Since the goal of the study was to regress recorded PPG features with the questionnaire outcome, this aspect poorly affected the findings, however it would be interesting to perform further studies to compare acute PPG recordings with STAI questionnaires performed either before or after the actual measurements.

The disadvantage of employing PPG for the evaluation of the emotional state for human-robot interaction is associated to the use of optical sensors located on the skin. In general, a contact-less solution is desirable for this kind of application. Recent development in hardware and software technologies allowed to measure PPG in a contact-less manner, making this technique highly suitable for emotion recognition and social robots applications (Wieringa, Mastik & Van der Steen, 2005; Wong, Pickwell-MacPherson & Zhang, 2010; Maestre-Rendon et al., 2020).

It is worth to notice that, although 66% of the SA explained variance might not seem extremely high, indeed this novel approach might be used in situation where a “real-time” feedback is needed or where performing a questionnaire is unfeasible (e.g., in BCI, in robot-human interaction, in assisted driving). Moreover, it should be further remarked that the SA scale is generally used in a coarse interpretation manner, for example dividing the scale in “low” and “high” anxiety level (Addolorato et al., 1999). This aspect makes the high performance of the binary classification of SA from PPG extremely relevant, and this high classification performance is directly connected to the amount of explained variance of SA using the proposed method.

Another aspect that is important to highlight is that general psychophysiological arousal or sympathovagal valence can affect the PPG signal since they affect the circulatory system (Mauri et al., 2010). However, estimating all the emotional factors that could affect the PPG signal is beyond the scope of this article. STAI-Y was used since it is indicative of the state anxiety of the participants and there is no evidence that STAI scales are multidimensional in terms of item content (Vagg, Spielberger & O’Hearn, 1980). Moreover, the anxiety stimulation allowed to test the capability of the model based on PPG features to estimate high levels of state anxiety.

Finally, it would be worth noticing that PPG is often employed for the arterial stiffness assessment, for instance relying on the estimation of PWV. However, given the strong association between psychophysiological condition and PPG, it is plausible that the arterial stiffness assessment can be influenced by the emotional condition during the examination, hence not providing an accurate result if the patient is stressed. The relationship between pathological condition of stress and arterial stiffness has already been investigated by several studies (Nomura et al., 2005; Logan et al., 2012), but the influence of the contingent psychophysiological status on the stiffness metrics evaluation needs further investigation.

Photoplethysmography, together with a machine learning approach, could provide concurrent information about the arterial status and the psychophysiological condition of patients, paving the way to innovative method of monitoring the patients’ emotional state in clinical environments (e.g., intensive care unit), and to assistive social robots that can interact with the patients thanks to the emotion recognition.

It is important to highlight that the sample population of the study is composed of participants without clinical issues related to cardiocirculatory or psychiatric disorders, hence the findings refer to the estimation of SA in healthy subjects through PPG features and a machine learning approach. The validity of the method also for diseased populations needs to be investigated in further studies.

## Conclusions

In this article, the relationship between SA and physiological parameters assessed by means of PPG in healthy people was investigated. The results confirmed that the SA could be predicted from brachial and ulnar PPG recordings by means of a multivariate machine learning approach. This novel method, together with improvements in PPG technology that allows to measure the signal at a distance, could pave the way to social robots able to assist patients and interact with them recognizing their emotions.

## Supplemental Information

10.7717/peerj.10448/supp-1 Supplemental Information 1 State Anxiety evaluated from STAI-Y.

10.7717/peerj.10448/supp-2 Supplemental Information 2 Regressors evaluated from photoplethysmography employed for the estimation of State Anxiety.

## Additional Information and Declarations

### Data Availability

The following information was supplied regarding data availability: The raw data, including regressors used in the model and variables used to train the model, are available in the Supplemental Files.
